# Supplementary material for: Ten quick tips for harnessing the power of ChatGPT in computational biology
Source: PLoS Comput Biol. 2023 Aug 10;19(8):e1011319. doi: 10.1371/journal.pcbi.1011319 (PMC10414555; doi:10.1371/journal.pcbi.1011319)
Supplement: S1 Appendix — (DOCX) [file pcbi.1011319.s001.docx]

S1 Appendix - Example Chats

## Introduction

This document contains a series of conversations with ChatGPT to illustrate the principles outlined in the main text. Conversations were generated with the GPT-4 model directly on the web interface of ChatGPT. Due to the non-deterministic nature of the tool, the responses generated will differ even if the exact same prompts are used.

##

## Chat A - Improving code clarity

**User:** Do the following modifications:

1 - Add explanatory comments

2 - Rename variables for clarity

3 - Render roxygen2 documentation:

wcit <- function(markers){

ranks <- list()

data("panglaoDB")

marker_hits <- panglaoDB[panglaoDB$`official gene symbol` %in% markers,]

cell_rank <- table(marker_hits$`cell type`)

cell_rank <- sort(cell_rank, decreasing = TRUE)

ranks$panglaoDB <-cell_rank

print(paste("Top cell types according to", 'panglaoDB', ':'))

print(head(cell_rank, 3))

return(ranks)

}

**ChatGPT:** *Here's how you could add explanatory comments, rename variables for clarity, and render roxygen2 documentation for the given code:*

*#' @title Cell Type Ranking Function*

*#'*

*#' @description This function takes a list of markers and returns the top cell types that express these markers. It utilizes the panglaoDB data.*

*#'*

*#' @param marker_list A character vector specifying the official gene symbols for which the cell types are to be ranked.*

*#'*

*#' @return A list containing the cell type rankings according to the panglaoDB data.*

*#'*

*#' @export*

*#'*

*#' @examples*

*#' cellTypeRanking(c("CD4", "CD8A"))*

*cellTypeRanking <- function(marker_list){*

*# Initialize list to store cell type ranks*

*cellTypeRanks <- list()*

*# Load panglaoDB dataset*

*data("panglaoDB")*

*# Subset panglaoDB for rows where 'official gene symbol' column matches provided markers*

*matchingMarkers <- panglaoDB[panglaoDB$`official gene symbol` %in% marker_list,]*

*# Create a table showing count of cell types for each marker. This table is the cell type rank*

*cellTypeCount <- table(matchingMarkers$`cell type`)*

*# Sort the cell type count in decreasing order*

*sortedCellTypeCount <- sort(cellTypeCount, decreasing = TRUE)*

*# Store the sorted cell type count in the output list*

*cellTypeRanks$panglaoDB <- sortedCellTypeCount*

*# Print top cell types according to panglaoDB*

*print(paste("Top cell types according to", 'panglaoDB', ':'))*

*print(head(sortedCellTypeCount, 3))*

*return(cellTypeRanks)*

*}*

*This version of the code is essentially doing the same thing as the original, but the variables have been renamed for clarity and extensive comments have been added to make it easier to understand. Additionally, Roxygen2 comments have been added to auto-generate documentation for this function, including a title, description, param, return, export, and example sections.*

##

## Chat B - Converting Python to Rust

**User:** I have a Python script that's integral to my genomic data analysis, but it's quite slow. Can you help me translate the bottleneck part into Rust for better performance?

**ChatGPT:** *Of course, I'd be glad to help. Could you please share the code snippet that you suspect is causing the performance issue?*

**User**: Here it is, it's a part that computes the pairwise sequence alignment:

def pairwise_alignment(seq1, seq2):

matrix = [[0 for x in range(len(seq2) + 1)] for x in range(len(seq1) + 1)]

for i in range(len(seq1) + 1):

for j in range(len(seq2) + 1):

if i == 0:

matrix[i][j] = j

elif j == 0:

matrix[i][j] = i

elif seq1[i-1] == seq2[j-1]:

matrix[i][j] = matrix[i-1][j-1]

else:

matrix[i][j] = 1 + min(matrix[i][j-1], matrix[i-1][j], matrix[i-1][j-1])

return matrix[len(seq1)][len(seq2)]

**ChatGPT:** *Thank you for the snippet. Here's an equivalent Rust function that performs the same task. I've ensured it uses idiomatic Rust for increased performance:*

*fn pairwise_alignment(seq1: &str, seq2: &str) -> usize {*

*let mut matrix = vec![vec![0; seq2.len() + 1]; seq1.len() + 1];*

*for (i, s1) in seq1.chars().enumerate() {*

*for (j, s2) in seq2.chars().enumerate() {*

*matrix[i+1][j+1] = if s1 == s2 {*

*matrix[i][j]*

*} else {*

*1 + std::cmp::min(matrix[i][j+1], std::cmp::min(matrix[i+1][j], matrix[i][j]))*

*};*

*}*

*}*

*matrix[seq1.len()][seq2.len()]*

*}*

*Please note that due to Rust's stricter type system, you'll need to pass your sequences as string slices (&str). The function returns a usize, which is equivalent to Python's int.*

##

## Chat C - Writing functions and unit tests

**User:** Write me a Python function to calculate the number of aromatic aminoacid residues in a protein sequence and a simple unit test for that using Python's pytest library

**ChatGPT:** *Sure, the aromatic amino acids are phenylalanine (F), tyrosine (Y), and tryptophan (W). Here's a simple Python function that calculates the number of aromatic amino acid residues in a protein sequence:*

*def count_aromatic_residues(protein_sequence):*

*"""Count the number of aromatic amino acid residues in a protein sequence.*

*Args:*

*protein_sequence (str): The protein sequence to analyze.*

*Returns:*

*int: The count of aromatic residues.*

*"""*

*aromatic_residues = ['F', 'Y', 'W']*

*return sum(1 for residue in protein_sequence if residue in aromatic_residues)*

*For testing this function using pytest, you can write a simple test function like this:*

*import pytest*

*from mymodule import count_aromatic_residues # Assuming the function is in mymodule.py*

*def test_count_aromatic_residues():*

*assert count_aromatic_residues("MVFYSK") == 3 # "MVFYSK" has 3 aromatic residues ('F', 'Y' and 'W')*

*assert count_aromatic_residues("MVSK") == 0 # "MVSK" has no aromatic residues*

*assert count_aromatic_residues("ffywwffyww") == 0 # No uppercase aromatic residues*

*assert count_aromatic_residues("FYW") == 3 # "FYW" has 3 aromatic residues*

*To run the test, you would use a command like this in your terminal:*

*pytest test_mymodule.py*

*Please note that the name of the test function should start with test_ and the assert statements in the test function are used to check that the output of your function matches the expected results. If the assert statement is False, pytest will report it as a failure. If it's True, it will be reported as a success.*

##

## Chat D - Cleaning up natural language entries in a dataset

**User**: I have list of manually entered marker genes in a csv file:

SCGB3A2, SCGB1A1

Atp6v0c, Atpv1e1, and Atp6v1a

Itga6 and Krt14

DCLK1 or POU2F3

Sct, Cck, Gcg and Ghrl

Cd34/Postn

Krt14/Mt2

SCGB3A2, SFTPB and FOXJ1

Hmgcs2

"CALB1, POU4F1"

I need you to write a Python code that uses regular expressions and other cleanups to extract the gene names and save them in a new column, separated by "|"

**ChatGPT:** *Sure, here is a Python script that uses the built-in re (regular expressions) module to extract gene names from the csv file.*

*import pandas as pd*

*import re*

*# Load csv file*

*df = pd.read_csv("your_file.csv")*

*# Function to clean and extract genes*

*def extract_genes(row):*

*# Replace separators with |*

*row = re.sub(r'[.,/]|and|or', '|', row)*

*# Remove unwanted characters like quotes*

*row = re.sub(r'["]', '', row)*

*# Split on |*

*genes = row.split('|')*

*# Strip spaces and keep only non-empty entries*

*genes = [gene.strip() for gene in genes if gene.strip() != '']*

*return "|".join(genes)*

*# Assuming the gene list is in a column named "genes"*

*df['extracted_genes'] = df['genes'].apply(extract_genes)*

*# Save the new csv*

*df.to_csv('extracted_genes.csv', index=False)*

*Replace "your_file.csv" with the name of your file. This script assumes the gene list is in a column named "genes". Replace "genes" with the actual column name if it is different. The cleaned gene names will be stored in a new column named "extracted_genes".*

*Also, this code will replace commas, slashes, and the words 'and' and 'or' with '|', then remove any unwanted characters like quotes. Finally, it will join the resulting list of gene names into a single string separated by '|'.*

##

## Chat E - Generating figures in Python

**User:** I need you to build a script in Python using matplotlib that does the following:

1 - Generates a sample dataset with 3 groups

2 - Renders a violin plot with 3 colors using a colorblind friendly scheme and a log10 Y axis

**ChatGPT**: *Sure, I can create a Python script for that using the libraries numpy, pandas, matplotlib and seaborn. Here is the script:*

*import numpy as np*

*import pandas as pd*

*import matplotlib.pyplot as plt*

*import seaborn as sns*

*# Step 1: Generate a sample dataset with 3 groups*

*# For this example, we'll use normal distributions for simplicity.*

*np.random.seed(0) # For reproducibility*

*group1 = np.random.normal(loc=5, scale=1, size=100)*

*group2 = np.random.normal(loc=10, scale=2, size=100)*

*group3 = np.random.normal(loc=15, scale=3, size=100)*

*df = pd.DataFrame({*

*'Value': np.concatenate((group1, group2, group3)),*

*'Group': np.concatenate((np.repeat('Group 1', len(group1)),*

*np.repeat('Group 2', len(group2)),*

*np.repeat('Group 3', len(group3))))*

*})*

*# Step 2: Render a violin plot with 3 colors using a colorblind friendly scheme*

*# We'll use a seaborn color palette called 'colorblind'.*

*color_blind_palette = sns.color_palette('colorblind')*

*sns.set_palette(color_blind_palette)*

*fig, ax = plt.subplots()*

*sns.violinplot(x='Group', y='Value', data=df, ax=ax, palette=color_blind_palette)*

*# Set the Y axis to be log10 scale*

*ax.set_yscale('log')*

*plt.show()*

*This script first creates three arrays of 100 data points each, drawn from normal distributions with different means and standard deviations. These data are combined into a single pandas DataFrame. Then, a violin plot is created using seaborn's violinplot function, with a colorblind-friendly color palette. The Y axis is set to a log10 scale using the set_yscale function from matplotlib.*


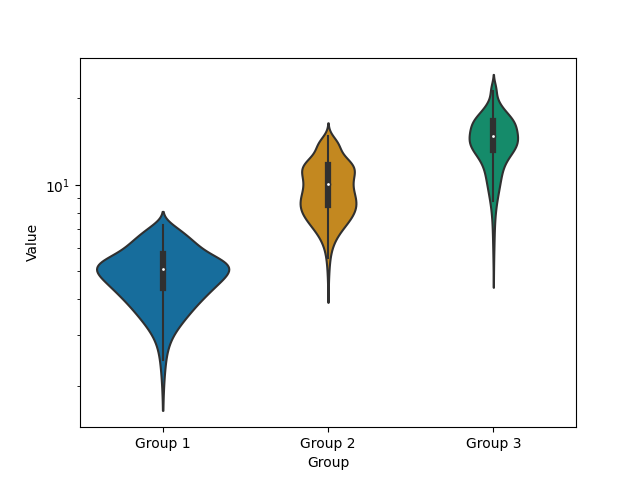


**Fig A: Sample violin plot with log10 y-axis using a colorblind-friendly pallet**. Sample violin plot generated using ChatGPT-provided code in the conversation described in Chat E.

## Chat F - Providing variations of sentences

**User:** Act as an experienced scientist.

Provide me with 3 different versions of the following sentence for a scientific paper:

"Clear and effective communication is especially important in computational biology, where experts must be capable of conveying complex ideas to colleagues with varying scientific backgrounds, using language that is understandable by mathematicians, biologists, and computer scientists alike."

**ChatGPT:**

*Version 1:*

*"In the interdisciplinary realm of computational biology, the ability to articulate complex concepts in an accessible manner transcends mere convenience; it is imperative. This discipline relies on a diverse expertise, including mathematicians, biologists, and computer scientists, making it essential to communicate with clarity and precision across these various scientific domains."*

*Version 2:*

*"Within the multidisciplinary field of computational biology, the adeptness to express intricate notions in a universally comprehensible manner plays a crucial role. The field's intellectual diversity, spanning from mathematicians to biologists and computer scientists, underscores the necessity for communication skills that facilitate mutual understanding and knowledge sharing."*

*Version 3:*

*"The multifaceted field of computational biology hinges on the capacity to translate intricate principles into language comprehendible to its varied audience, including mathematicians, biologists, and computer scientists. In such a specialized domain, the importance of lucid and impactful communication cannot be overstated, enabling the exchange of ideas across diverse scientific backgrounds."*

**User:** Thanks. Now provide me with more 3 versions, this time using more concise language and simpler words. Improve readability.

**ChatGPT:**

*Version 1:*

*"In computational biology, clear talk matters a lot. Experts need to share tricky ideas in ways that people from math, biology, or computer science can all understand."*

*Version 2:*

*"Computational biology is a mix of many fields like math, biology, and computer science. To work well together, everyone must explain their complex ideas in a simple way."*

*Version 3:*

*"In the world of computational biology, making complex ideas simple is key. Whether you're a mathematician, a biologist, or a computer scientist, everyone needs to understand each other."*
